# The Ameliorative Role of *Acacia senegal* Gum against the Oxidative Stress and Genotoxicity Induced by the Radiographic Contrast Medium (Ioxitalamate) in Albino Rats

**DOI:** 10.3390/antiox10020221

**Published:** 2021-02-02

**Authors:** Islam El-Garawani, Sobhy Hassab El-Nabi, Ahmed El Kattan, Azza Sallam, Sabha Elballat, Shaimaa Abou-Ghanima, Islam H. El Azab, Hesham R. El-Seedi, Shaden A. M. Khalifa, Sawsan El-Shamy

**Affiliations:** 1 Zoology Department, Faculty of Science, Menoufia University, Shebin El-Kom, Menoufia 32511, Egypt; drsobhyhassabelnabi@science.menofia.edu.eg (S.H.E.-N.); ohoodsallam@science.menofia.edu.eg (A.S.); 2 Medical and Radiation Department, Researcher Sector, Nuclear Materials Authority, Cairo 11381, Egypt; kattan.a79@nma.org.eg; 3 Zoology Department, Faculty of Science, Zagazig University, Zagazig 44519, Egypt; sabhael@zu.edu.eg; 4 Biochemistry Department, King Khalid University, Abha 61421, Saudi Arabia; sjamal@kku.edu.sa; 5 Chemistry Department, College of Science, Taif University, P.O. Box 11099, Taif 21944, Saudi Arabia; i.helmy@tu.edu.sa; 6 Department of Molecular Biosciences, The Wenner-Gren Institute, Stockholm University, S-10691 Stockholm, Sweden; shaden.khalifa@su.se; 7 International Research Center for Food Nutrition and Safety, Jiangsu University, Zhenjiang 212013, China; 8 Chemistry Department, Faculty of Science, Menoufia University, Shebin El-Kom, Menoufia 32511, Egypt; 9 Basic Science Center, Department of Biology, Misr University for Science and Technology, Giza 12511, Egypt; sawsan.alshamy@must.edu.eg

**Keywords:** Arabic gum, DNA damage, genotoxicity, Ioxitalamate, oxidative stress, LC-MS-MS

## Abstract

Arabic gum (*Acacia senegal*, AG) is proven effective antioxidant and cytoprotective agent. The present study was designed to test this notion by investigating the possible role of AG against the radiographic contrast medium (Ioxitalamate, Telebrix-35^®^, TBX)-induced oxidative stress and genotoxicity. Albino rats were divided into four groups and supplied with either; distilled water, daily 10% (*w*/*v*) AG, an intravenous dose of TBX (1600 mg I/kg b.wt) and co-administration of TBX and AG. Rats were sacrificed and blood samples were collected to assess the genotoxicity employing the peripheral blood leucocytes fluorescent double staining; namely the acridine orange/ethidium bromide (AO/EB) staining and alkaline comet assay. Further, chromosomal analyses were done in bone marrow cells. Serum urea and creatinine levels, in addition to malondialdehyde (MDA), nitric oxide (NO), catalase (CAT) and glutathione (GSH) levels in kidney tissues were measured. Liquid chromatography-mass spectrophotometry (LC-MS-MS) was performed to identify the chemical composition of AG extract. Kidney functions, single/double-stranded DNA damage, chromosomal aberrations, mitotic index, MDA and NO levels were significantly (*p* < 0.001) increased in TBX-treated group compared to the control and AG-treated one. Meanwhile, CAT and GSH activities were significantly diminished and the AG supplementation significantly (*p* < 0.001) ameliorated these effects compared with the control and AG-treated groups. Five compounds have been identified using GNPS networking including 7,3′,4′-Trihydroxyisoflavone, Noscapine, Tetrahydropapaveroline, Costunolide, Hesperidin. In conclusion, results of the present study suggest that AG exerted a protective role against TBX-induced oxidative stress and genotoxicity which may be attributed to the active metabolites in the gum.

## 1. Introduction

Contrast media (CM) is a common group of chemicals commonly used in radiographic diagnosis and radiotherapy [1,2]. Iodine is a crucial element used in CM due to its high-contrast density [3]. It is a tri-iodinated derivative of benzoic acid [4]. Iodinated contrast agents have different osmolality [5]. The higher CM osmolality, the severe adverse effects [6]. For instance, hyperthyroidism or hypothyroidism may be correlated to the iodinated CM exposure. Moreover, nephropathy is certainly the most common adverse effect [3].

Ioxitalamate (Telebrix^®^), is an ionic iodinated CM widely used for radiography [7]. The iodinated radiographic CM-induced nephropathy is associated with renal apoptosis and oxidative stress [8,9,10]. Cytogenetic changes in bone marrow cells were reported after CM administrations in mice [1], manifested by the increase of micronucleated polychromatic erythrocytes [11].

Arabic gum (AG) is an edible, dried gummy exudate from the stems and branches of *Acacia senegal* tree. It is characterized by the richness of non-viscous soluble fibers. It is commonly used as an emulsifier and stabilizer in the food and pharmaceutical industries [12]. Among other rich natural products, Arabic gum has a history as a preventive supplementation in Arabic folk medicine and recently attracted more attention due to its protective impact against the effects of environmental and chemical exposure to hazards [13,14,15,16,17,18,19].

The high-molecular weight polysaccharides and their potassium, magnesium and calcium salts are the major constituents of AG. After AG hydrolysis, arabinogalactan, arabinogalactanprotein and glycoprotein were the main fractions obtained [20]. AG is considered a safe food additive as it did not induce genotoxicity or carcinogenicity when given intraperitoneally or orally [21]. Arabic gum plays an important antioxidant and protective role against experimental gentamicin and cisplatin-induced nephrotoxicity [22], doxorubicin cardiotoxicity [23] and acetaminophen hepatotoxicity [24]. It is also known to combat many diseases such as renal [12], cardiovascular [25], gastrointestinal [26] and respiratory diseases, due to its agonist effect on the oxidative stress and DNA damage [27]. Moreover, Badreldin et al. [28] confirmed the antioxidative and anti-inflammatory properties of AG. The antischistosomal and protective effects on some reproductive aspects in mice were also reported [29]. Clinically, in chronic renal failure patients, AG has been shown to be beneficial [30]. 

Thus, the present investigation aimed to evaluate the protective potentials of Arabic gum against the iodinated radiographic contrast medium (Ioxitalamate)-induced genotoxicity and oxidative stress in rats.

## 2. Materials and Methods

### 2.1. Chemicals

Radiographic contrast medium, Ioxitalamate (C_12_H_11_I_3_N_2_O_5_); [CAS. 20978/00, Lyomit, Telebrix-35^®^, GASTRO, Guerbet, France] was enrolled in the current study. Agarose gel (Sigma-Aldrich, Darmstadt, Germany), DNA ladder (100 bp, O’gene ruler™, Thermo Fisher Scientific, Austin, TX, USA), normal melting point ultra-pure agarose (Sigma-Aldrich, Darmstadt, Germany) and ethidium bromide (Sigma-Aldrich, Darmstadt, Germany).

### 2.2. Arabic Gum

It is a soluble dietary fiber obtained naturally from the stems and branches of *Acacia senegal* trees (family: legume). It was purchased from El-Nobi Co. (Shebin El-Kom, Menoufia, Egypt) as crystals (commercial grade) and ground to a fine powder. An aqueous solution of Arabic Gum (10%, *w*/*v*) was prepared freshly every day and introduced to the rats in drinking water [31,32].

### 2.3. Experimental Animals

The study was carried out on apparently healthy 60 adult Wister male albino rats, weighing 180 ± 15 g (7–9 weeks old), obtained from the Holding Company for Biological Products & Vaccines (VACSERA), Egypt. Animals were housed in cages in a controlled animal facility environment (room temperature about 25 °C, 12 h light and 12 h dark cycle), with free access to a standard commercial diet (El-Haramain^®^ standard rodent diet, Egypt) and water was *ad libitum*. Animals were acclimatized for 14 days prior to the experiment. The study followed the Institutional Animal Ethical Committee (IAEC) guidelines at the Faculty of Science, Menoufia University (MUFS-F-GE-2-20).

### 2.4. Experimental Design

Animals were divided into four groups (15 rats/group). Group (1) animals served as a negative control group, Group (2) animals received 10% (*w*/*v*) of AG in drinking water daily, Group (3) animals were subjected to the codal vein, intravenous, single dose injection (1600 mg I/kg b.wt., equivalent to the higher range of the human dose) according to Li et al. [33] and Group (4) animals were injected with Telebrix (single dose, i.v.) and received 10% (*w*/*v*) of AG daily. Five rats per group, randomly selected, were sacrificed after 1 day (for liver and peripheral leucocytes analyses) and the other 5 rats/group were sacrificed after 14 days for liver sampling only.

In addition, further genotoxicity investigation was done in parallel using 5 rats/group which processed for bone marrow chromosomal preparations after 24 h of treatments.

### 2.5. General Health

All rats were monitored to assess mortality, skin irritation, food intake and activities during the period of the experiment.

### 2.6. Kidney Functions

Sera of treated and control rats were collected, by centrifugation at 900× *g* for 5 min, from submandibular peripheral venous blood samples (2 mL/rat) after clotting. Urea and creatinine levels were determined according to the method of Tabacco et al. [34] and Rartels and Böhmer [35] using Milton Roy spectrophotometer (Spectronic 1201, Houston, TX, USA).

### 2.7. Oxidative Status in Kidneys’ Tissue

Kidney tissues homogenate (10%, *wt*/*v*) were prepared in phosphate buffer (0.1 M, pH 7.4). Supernatants were collected by centrifugation at 3600× *g* for 5 min and kept at −20 °C for further investigations. 

Catalase activities were measured according to the method of Cohen et al. [36]. Lipid Peroxidation (MDA) was determined at 534 nm using the method of Mesbah et al. [37] The sulfhydryl group of GSH was measured colorimetrically at 412 nm according to Ellman [38]. Nitric oxide levels were determined following the procedure of Green et al. [39]. All investigations were performed using Milton Roy spectrophotometer (Spectronic 1201, Houston, TX, USA)

### 2.8. Total Genomic DNA Extraction and Apoptosis Detection in Kidney Tissue

DNA extraction and detection of apoptosis (DNA fragmentation assay) were done according to the salting out extraction method of Aljanabi and Martinez [40] and modifications by El-Nabi and Elhassaneen [41]. About 20 mg of tissue samples from treated and control kidneys were lysed in lysing buffer at 40 °C for 24 h. Then, proteins were precipitated by a solution of NaCl (4 M). Cold isopropanol was used for nucleic acids precipitation. The resuspended pellets of nucleic acids in Tris-EDTA buffer (10 mM Tris-HCl, 1 mM EDTA, pH 8) were incubated for 60 min. with a loading buffer supplemented with RNase. The samples were stained directly with ethidium bromide [42] and processed for electrophoresis on 1.8% agarose gel. Apoptotic bands of DNA were aligned, at 180 bp and its multiples 360, 540 and 720 bp, against 100 bp DNA ladder. The intensity of released DNA fragments was analyzed using Image J software at 256 grey levels. 

### 2.9. Isolation of Peripheral Blood Leucocytes 

Heparinized peripheral venous blood samples (2 mL/rat) were drawn from treated and control animals. Then leucocytes were isolated from rats’ peripheral blood by incubation (1:3, *v*/*v*) with erythrocyte lysing buffer [NH_4_C1 (0.015 M), NaHCO_3_ (1 mM), EDTA (0.l mM)]. They were centrifuged for 5 min at 900× *g*. These steps were repeated until a white pellet of leucocytes appeared [43]. Then, the pellets were resuspended in RPMI 1640 medium for further investigations. 

### 2.10. Leucocytes Double Staining by Acridine Orange/Ethidium Bromide (AO/EB)

One microliter of florescent dye mixture (100 mg/mL of AO and 100 mg/mL of EB in distilled water) was incubated with 4 µL of leucocytes’ suspension for 1 min. On a microscopic glass slide, the suspension was directly examined by a fluorescent microscope (Olympus CX23, Tokyo, Japan) at 400× magnification. A minimum of 400 cells were counted per a sample [44].

### 2.11. Alkaline Comet Assay in Peripheral Blood Leucocytes

In the present study, alkaline single cell gel electrophoresis (comet assay) was performed to assess the DNA damage in peripheral blood leucocytes of untreated and treated rats after 24 h [45]. Briefly, the mixed cells with low melting point agarose gel were layered between two layers of ultra-pure agarose with a normal melting point, on microscopic glass slides. In dark conditions, the slides were dipped in lysis buffer (2.5 M NaCl, 100 mM EDTA and 10 mM Tris, 1% Triton X-100, 10% DMSO, pH 10) for 1 h at 4 °C. The slides were kept in a cold and freshly prepared alkaline buffer (300 mM NaOH and 1 mM EDTA, pH > 13) for 10 min. Then, they were subjected to 25 V/300 mA electric current for 15 min. Neutralization of the slides was carried out using 0.4 M Tris-HCl buffer, pH 7.5 three times by rinsing. Next, they were labeled with ethidium bromide (20 μg/mL). Nuclei were visualized using Olympus, fluorescence microscope (BX41, Tokyo, Japan). For DNA damage assessment, about 50 randomly selected nuclei were inspected per field of total examined five fields per slide. The results were visually classified as normal nuclei with no tail, lightly damaged with a migrated tail less than the nucleus diameter and strongly damaged with a DNA migration more than the nucleus diameter.

### 2.12. Bone Marrow Chromosomal Preparations

Chromosomal preparations were carried out in bone marrow of treated and control rats [46]. Animals were injected 2 h before sacrificing with 0.2 mL (0.0012%) colchicine to arrest the cell division at metaphase. Briefly, bone marrow cells were collected from the femurs in isotonic solution (0.9% NaCl). Hypotonic solution (0.56% KCl) was added to the cells and they were incubated for 20 min at 37 °C. Then, cells were drop-wise fixed by 3–4 mL of cold and freshly prepared fixative (absolute methanol and glacial acetic acid, 3:1, *v*/*v*) for 15 min. This step was repeated three times to complete fixation. Then, the pellets were re-suspended in 100 µL of the fixative and dropped on a clean slide previously dipped in cold 70% ethyl alcohol then air-dried after flaming. Slides were stained with phosphate-buffered Giemsa solution. For each animal, 100 well spread metaphases were scored for chromosomal aberrations at 1000× magnification using a light microscope (Olympus CX23, Tokyo, Japan). Furthermore, the assessment of the mitotic index was carried out by evaluating five hundred cells per group at 400× magnifications using a light microscope (Olympus CX23, Tokyo, Japan). The mitotic index was calculated using the following equation: 

Mitotic index (%) = (No. of metaphases + prophases)× 100/(No. of all counted nuclei).

The abnormal metaphases were evaluated also in the same slides and the calculations were carried out using the following equation: 

Abnormal metaphases (%) = (No. of abnormal metaphases) ×100/(No. of normal metaphases + No. of abnormal metaphases).

### 2.13. Extraction of Acacia senegal Gum

The raw material was grounded and soaked in DCM-MeOH 1:1 on shaker overnight and evaporated using rotatory evaporator. The residue was redissolved in cold water overnight and was dried using freeze dryer. The crude extracts were ready for further analysis.

### 2.14. LC-MS-MS Analysis

For LC-MS analysis, the crude extract was dissolved in 50% acetonitrile (ACN), 0.1% formic acid (FA). The samples were injected by syringe through a PicoTip emitter at 0.3 µL/min connected to a Q-Tof Micro (Waters, Milford, MA, USA) with the voltage set at 1.4 kV. The analysis was carried out in positive ion mode and linear gradient from 10% (*v/v*) H_2_O to 99% (*v/v*) ACN in 0.1% (*v/v*) FA at a flow rate of 0.3 µL/min over 75 min. The analysis was followed by LC/MS-MS where 0.1mg/mL in LC-MS solvent: 60% MeCN in 0.1% FA was dissolved with a linear gradient ranging from 10–60% (*v/v*) MeCN in 0.1% (*v/v*) FA at a flow rate of 0.3 mL/min over 75 min. The capillary temperature was set at 220 °C and the spray voltage at 4 kV [47]. GNPS molecular network analysis was performed to identify the metabolites [48,49].

### 2.15. Statistical Data Analysis 

All experiments were done in triplicates (*n* = 5). Data were presented as Mean ± SE. Using IBM SPSS software version 21.1 (New York, NY, USA), data were statistically analyzed and *p* < 0.001 were considered significant. 

## 3. Results

### 3.1. General Health

All treated and control rats showed no mortality during the period of the experiment. No irritation was observed at the TBX sites of injection.

### 3.2. Kidney Functions

**Figure 1:**
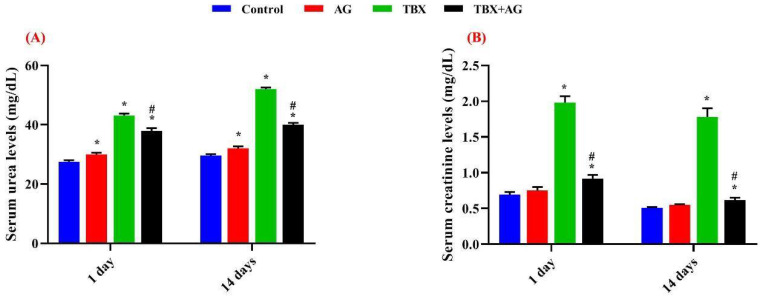
The protective effect of Arabic gum (*Acacia senegal*, AG) against the Telebrix (TBX)-induced alterations at the levels of serum urea (**A**) & creatinine (**B**) in treated rats after 1 and 14 days of the experiment. *: Significant (*p* < 0.001) relative to untreated groups. #: Significant (*p* < 0.001) relative to TBX-treated groups. Data were represented as mean ± SE of three independent experiments (*n* = 5). AG: 10% Arabic gum, TBX: Telebrix (1600 mg/kg b.wt.) and TBX+AG: co-treatment with 1600 mg/kg b.wt. and 10% Arabic gum.

In the present study, serum urea and creatinine were measured in the four groups. The administration of TBX induced a significant (*p* < 0.001) increase in serum urea and creatinine levels of treated rats with respect to the control. However, treatment with AG significantly (*p* < 0.001) ameliorated these effects. Serum urea levels were improved by ~11.6 & 23% in AG + TBX combined group relative to the TBX-treated group after 1 and 14 days of treatments respectively. Regarding serum creatinine, the improvements were ~53.5 & 65% in AG + TBX combined group relative to the TBX-treated group after 1 and 14 days of treatments respectively (Figure 1).

**Figure 2:**
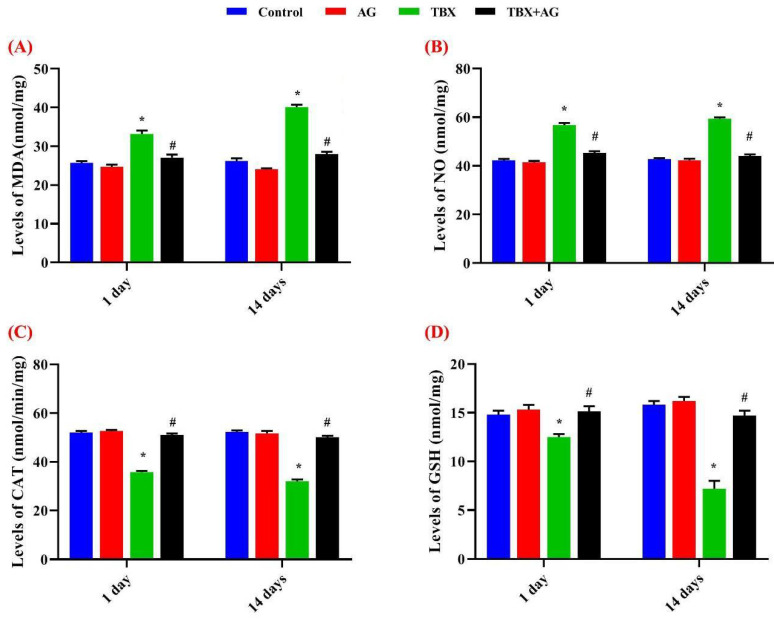
The protective effect of AG against TBX-induced alterations in oxidative stress markers after 1 and 14 days of treatments. (**A**) MDA: malondialdehyde, (**B**) NO: nitric oxide, (**C**) CAT: catalase and (**D**) GSH: glutathione. *: Significant (*p* < 0.001) relative to untreated groups. #: Significant (*p* < 0.001) relative to TBX-treated groups. Data were represented as mean ± SE of three independent experiments (*n* = 5). AG: 10% Arabic gum, TBX: Telebrix (1600 mg I/kg b.wt.) and TBX+AG: co-treatment with 1600 mg/kg b.wt. and 10% Arabic gum.

Following the treatments for 1 & 14 days, the oxidative markers were evaluated in the kidney tissue homogenates. Results revealed that TBX significantly (*p* < 0.001) elevated the lipid peroxidation (LPO), as measured by malondialdehyde (MDA) and nitric oxide (NO) concentrations (Figure 2A,B). Otherwise, a significant (*p* < 0.001) decrease in catalase (CAT) activities and glutathione (GSH) levels were observed as compared to control rats (Figure 2C,D). Whereas, rats in the AG+TBX combined group showed a significant amelioration exerted by AG against TBX effects after 1 and 14 days of treatments. The activities, after 1 and 14 days, of CAT and GSH were improved by about 41.6 & 56% and 20 & 100% respectively. However, LPO and NO levels were improved by approximately 18.4 & 30% and 21 & 26% after 1 and 14 days; respectively.

### 3.3. Genomic Double-Strand DNA Damage in Kidneys’ Tissue

**Figure 3:**
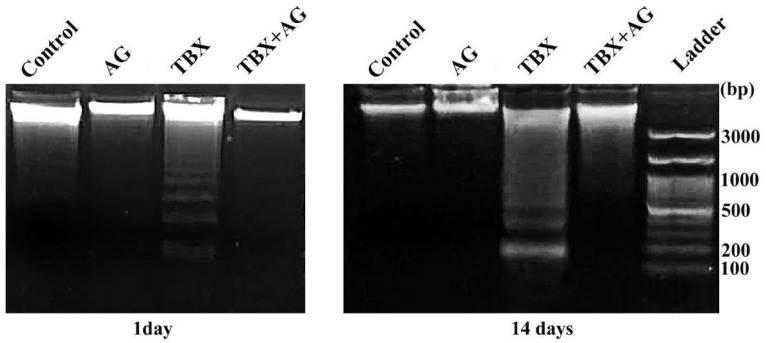
Representative photographs (1.8% ethidium bromide-stained agarose gel) show the protective effect of AG against TBX-induced DNA damage in kidney tissues after 1 and 14 days of treatment. AG: 10% Arabic gum, TBX: Telebrix (1600 mg I/kg b.wt.) and TBX+AG: co-treatment with 1600 mg/kg b.wt. and 10% Arabic gum.

**Figure 4:**
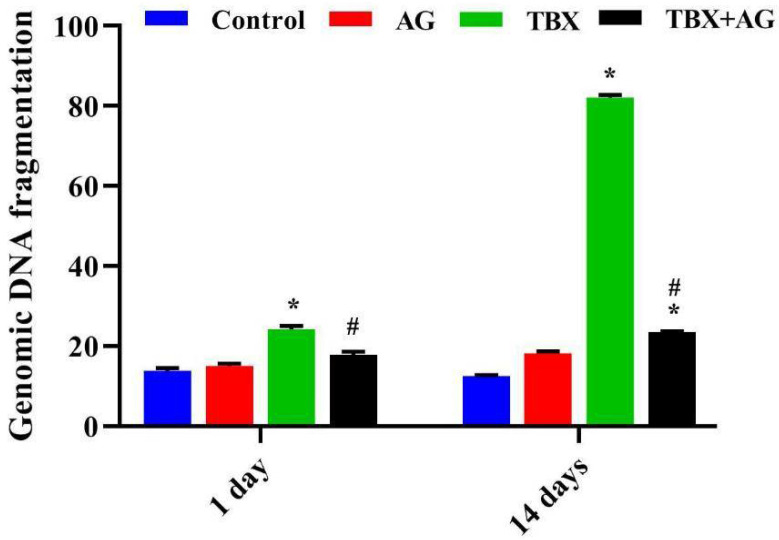
The protective effect of AG against TBX-induced DNA fragmentation employing 1.8% agarose gel electrophoresis of kidney tissues of treated rats after 1 and 14 days of treatments. *: Significant (*p* < 0.001) relative to untreated groups. #: Significant (*p* < 0.001) relative to TBX-treated groups. Data were represented as mean ± SE of three independent experiments (*n* = 5). AG: 10% Arabic gum, TBX: Telebrix (1600 mg I/kg b.wt.) and TBX+AG: co-treatment with 1600 mg/kg b.wt. and 10% Arabic gum.

The double-strand breaks were evaluated in kidneys’ tissue by agarose gel electrophoresis. TBX treatments induced internucleosomal cleavage as illustrated by the laddering pattern of DNA damage, a hallmark of apoptosis (Figure 3). A significant (*p* < 0.001) time-dependent increase in DNA fragmentation was recorded in the kidneys tissue of TBX-treated rats after 1 and 14 days when compared to control (Figure 4). Otherwise, in the case of AG combined treatments, a noticeable decrease in DNA damage (26 and 71.4%) was detected after 1 and 14 days of treatments respectively, when compared to TBX-treated rats.

### 3.4. Cytotoxicity on Leucocytes

**Figure 5:**
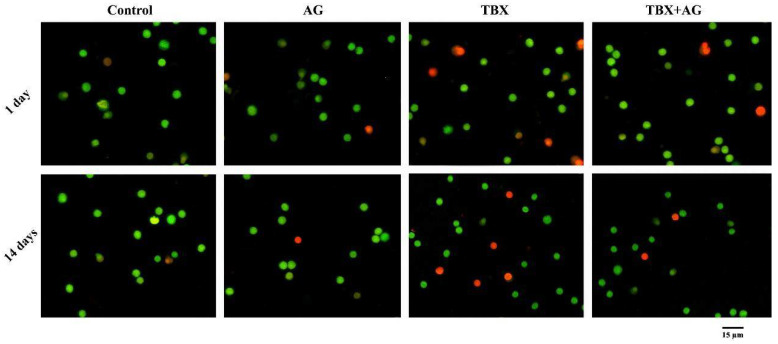
Representative photomicrographs show the protective effect of AG against TBX-induced cytotoxicity in peripheral blood leucocytes of treated rats after 1 and 14 days of the experiment. The isolated leucocytes were stained by AO/EB fluorescent dyes (Olympus CX23 microscope, Japan). AG: 10% Arabic gum, TBX: Telebrix (1600 mg/kg b.wt.) and TBX+AG: co-treatment with 1600 mg/kg b.wt. and 10% Arabic gum.

**Figure 6:**
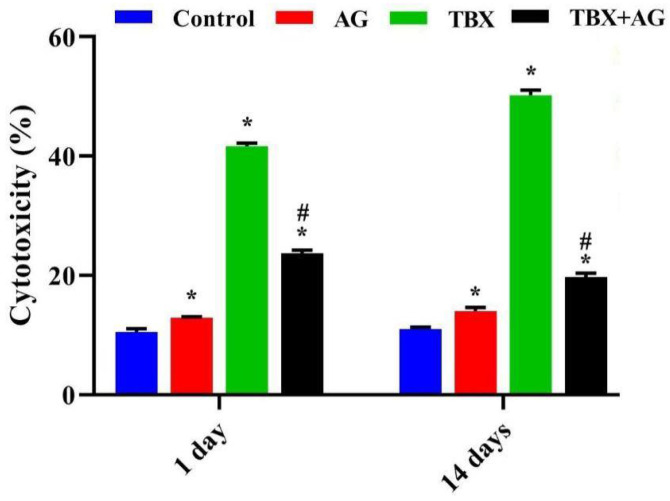
The protective effect of AG against TBX-induced alterations in the viability of peripheral blood leucocytes of treated rats after 1 and 14 days of the experiment. *: Significant (*p* < 0.001) relative to untreated groups. #: Significant (*p* < 0.001) relative to TBX-treated groups. Data were represented as mean ± SE of three independent experiments (*n* = 5). AG: 10% Arabic gum, TBX: Telebrix (1600 mg I/kg b.wt.) and TBX+AG: co-treatment with 1600 mg/kg b.wt. and 10% Arabic gum.

Results of AO/EB double fluorescent labeling (Figure 5) revealed a significant (*p* < 0.001) time-dependent elevation of the number of dead cells in TBX-treated rats after 1 and 14 days when compared to the untreated rats. Moreover, the combined treatment with AG showed a significant protective effect on leucocytes as the percentage of damaged cells was reduced by approximately 43% and 60% after 1 and 14 days, respectively (Figure 6).

### 3.5. Acute Genotoxicity in Leucocytes

#### 3.5.1. DNA Strand Breaks in Leucocytes

**Figure 7:**
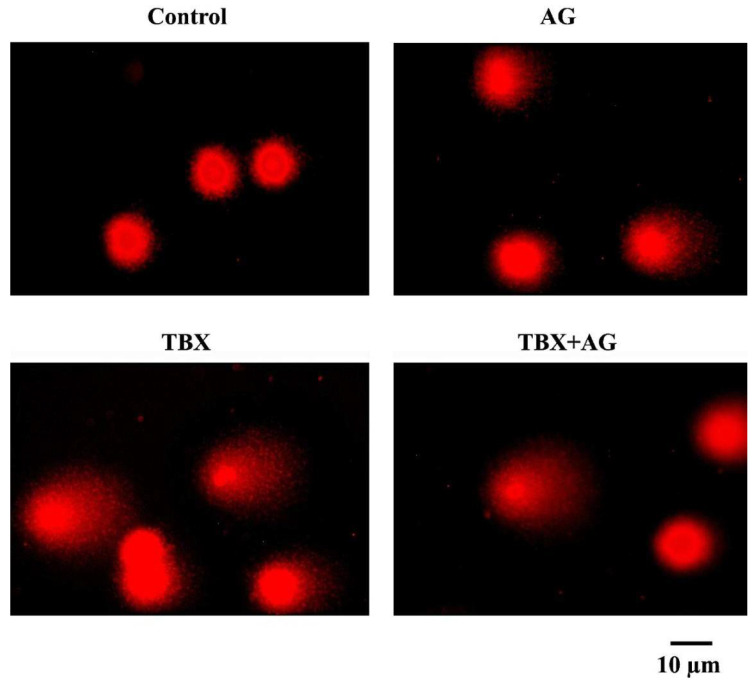
Representative photomicrographs of comet assay (ethidium bromide-stained) showing the protective effect of AG against TBX-induced DNA strand breaks in isolated peripheral blood leucocytes of treated rats after 24 h of treatments (Olympus CX23 microscope, Tokyo Japan). AG: 10% Arabic gum, TBX: Telebrix (1600 mg I/kg b.wt.) and TBX+AG: co-treatment with 1600 mg/kg b.wt. and 10% Arabic gum.

**Figure 8:**
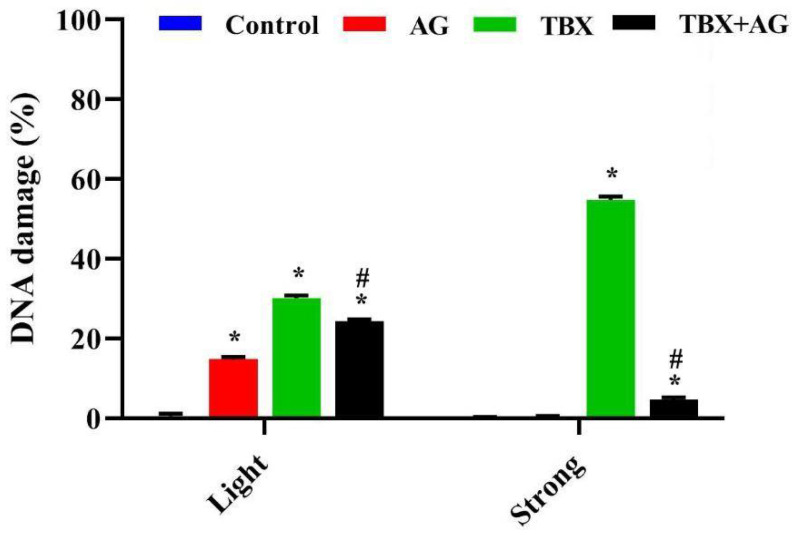
The protective effect of AG against TBX-induced DNA strand breaks in isolated peripheral blood leucocytes of treated rats after 24 h of treatments. *: Significant (*p* < 0.001) relative to untreated groups. #: Significant (*p* < 0.001) relative to TBX-treated groups. Data were represented as mean ± SE of three independent experiments (*n* = 5). AG: 10% Arabic gum, TBX: Telebrix (1600 mg I/kg b.wt.) and TBX+AG: co-treatment with 1600 mg/kg b.wt. and 10% Arabic gum.

DNA strand breaks were detected using single-cell gel electrophoresis (alkaline comet assay). The extent of DNA damage was evaluated as a length of DNA tail migration towards the anode. In the present work, TBX treatments for 24 h induced a significant (*p* < 0.001) DNA strand breaks in rats leucocytes as compared to controls (Figure 7). The light damaged cells were 30.1 ± 0.67% and the strong damaged cells were scored as 54.8 ± 0.8% (Figure 8). However, the combined treatment with AG showed significant (*p* < 0.001) improvements in DNA strand breaks as compared with TBX-treated rats (Figure 7). The detected light DNA damage was 24.26 ± 0.49 with about 20 improvement relative to the TBX-treated group. Whereas, the strong damage was 4.6 ± 0.6% with approximately 91% improvement when compared with TBX-treated group (Figure 8).

#### 3.5.2. Chromosomal Aberrations in Bone Marrow

**Figure 9:**
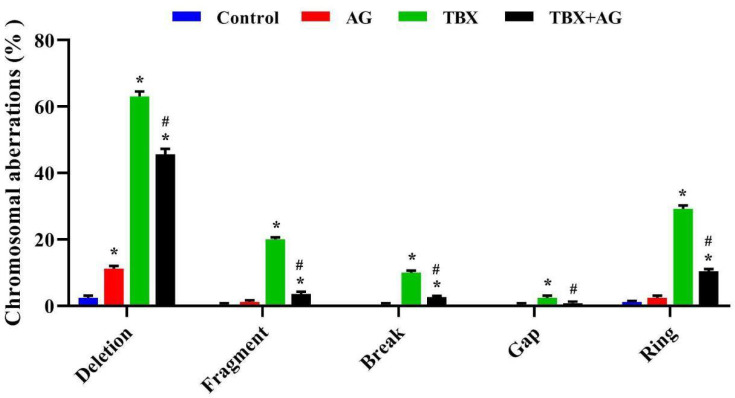
The protective effect of AG against TBX-induced chromosomal abnormalities in the bone marrow of treated rats after 24 h of treatments. *: Significant (*p* < 0.001) relative to untreated groups. #: Significant (*p* < 0.001) relative to TBX-treated groups. Data were represented as mean ± SE of three independent experiments (*n* = 5). AG: 10% Arabic gum, TBX: Telebrix (1600 mg I/kg b.wt.) and TBX+AG: co-treatment with 1600 mg/kg b.wt. and 10% Arabic gum.

In the current investigation, the structural chromosomal aberrations (deletion, fragmentation, breaking down or forming gap and ring) were scored in bone marrows among treated and untreated rats. Figure 9 showed a significant (*p* < 0.001) increase in the percentage of the evaluated aberrations among the TBX-treated rats where the total chromosomal aberrations without gaps was (TCA: 42 ± 0.7) with respect to the control (TCA: 0.6 ± 0.3). The highest recorded values were 63 ± 1.6% as detected as chromatid deletions and the lowest were gaps (2.4 ± 0.7%). However, the AG-treated animals revealed a significant (*p* < 0.001) decrease in the observed aberrations when compared to TBX-treated rats with approximately 69% improvement in TCA.

#### 3.5.3. Assessment of the Mitotic Index and Abnormal Metaphases

**Figure 10:**
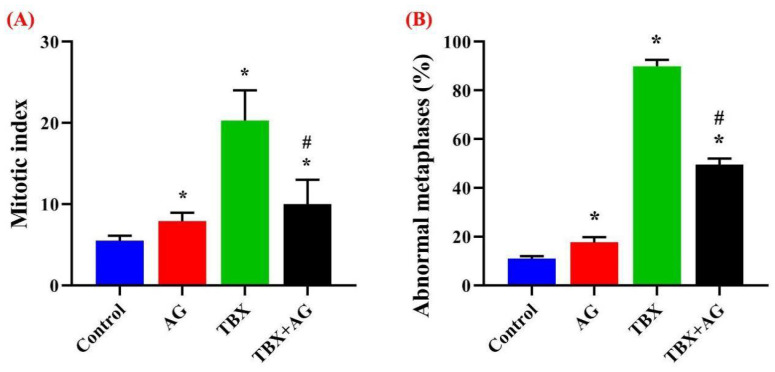
The protective effect of AG against TBX-induced mitotic index alterations (**A**) and the percentage of abnormal metaphases (**B**) in the bone marrow of treated rats after 24 h of treatments. *: Significant (*p* < 0.001) relative to untreated groups. #: Significant (*p* < 0.001) relative to TBX-treated groups. Data were represented as mean ± SE of three independent experiments (*n* = 5). AG: 10% Arabic gum, TBX: Telebrix (1600 mg I/kg b.wt.) and TBX+AG: co-treatment with 1600 mg/kg b.wt. and 10% Arabic gum.

In order to assess the proliferation capacities, chromosomal preparations were carried out in bone marrows of treated and untreated rats then, the mitotic indices were calculated. Surprisingly, TBX treatments caused a significant (*p* < 0.001) increase in the dividing populations (pro- and metaphases). While, AG administration significantly ameliorated, about 50%, of this elevation (Figure 10A). Moreover, the abnormal metaphase percentages were assessed along with the mitotic index evaluation. Figure 10B illustrated the significant (*p* < 0.001) increase in abnormal metaphases among TBX treatments whereas the protective potentials of AG combined treatments significantly (*p* < 0.001) restored these elevated levels by about 48%.

### 3.6. Chemical Investigation of Acacia senegal Extract

**Figure 11:**
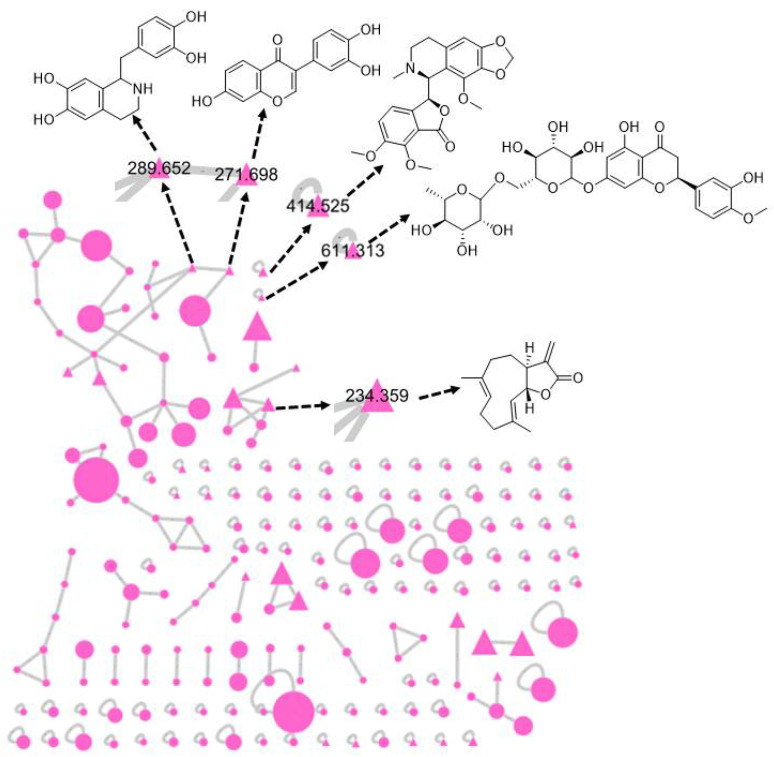
Molecular network of 195 parent ions produced from *A. senegal*. The circular fuchsia nodes refer to the whole parent masses that have unique detected peaks in the molecular network. The triangle fuchsia Nodes represent parent ions that have been identified in the GNPS molecular network.

The mass profile of *A. senegal* extract was analyzed to exhibit 195 nodes in the Global Natural Product Social Molecular Networking (GNPS), showed in Figure 11. All the metabolites detected in the raw mass file represented in the molecular network as nodes. The bigger the nodes the higher the intensity of their peaks in the raw mass file. The chemically related metabolites with similar common classes are clustered together. From 195 metabolites, only six were trustable when screened and compared with the previously isolated ones and found to have similar fragmentation patterns. One of the six identified metabolites was isolated previously from the same genus which was hesperidin. The five metabolites that were matched with other standards in the GNPS library, identified as 7,3′,4′-trihydroxyisoflavone, noscapine, tetrahydropapaveroline, costunolide and hesperidin. The precursor masses were as follows, [M + H]^+^ (*m*/*z*; 271.698, 414.525, 289.652, 234.359 and 611.313) as shown in Table 1.

## 4. Discussion

The present study was designed to assess the toxic effects of TBX (Ioxitalamate) on peripheral leucocytes and kidney tissues. It has thrown more light on the potential of AG treatments against the genotoxicity and oxidative stress induced by TBX in rats.

Ioxitalamate is an ionic iodinated contrast medium with a high osmolality of 1500–1800 mOsm/kg [55]. It has been shown to be associated with an incidence of nephrotoxicity [56] and directly damages the chemical bonds of DNA molecules [57]. However, radiation dose that presents in ioxitalamate contrast media may lead to the elevated number of DNA double-strand breaks [58,59]. Ionizing radiation has enough energy to release one or more electrons within biomolecules and subsequently change the chemistry of the DNA molecule leading to one or more single or double-strand DNA breaks [60]. Data of the present study showed that TBX induced DNA strand breaks in peripheral leucocytes and DNA double-strand internucleosomal cleavage in kidneys’ tissues. This was in agreement with the previous studies that illustrated that contrast medium caused in vivo [61,62,63] and in vitro [61,64] DNA damage. The intravenous injection with ioxitalamate in rats was claimed to lead to DNA damage and apoptosis [65], in vivo chromosomal aberrations [66] and micronuclei [67]. Similar to another study [68], the damage caused by radiocontrast agents may be related to their hyperosmolar cytotoxicity leading to apoptosis. However, Chromosomal damage in human somatic cells can trigger mutations and thus possibly the development of tumor cells and they can exert clastogenic effects [67]. The elevated mitotic index and proliferation in TBX-treated rats were seen previously prior to cancer development [69,70].

The biochemical and oxidative stress recorded in the current study showed a significant increase in the levels of serum urea and creatinine [71], the concentrations of lipid peroxidation and nitric oxide, accompanied with the decrease in catalase activities and glutathione concentrations in kidney tissues after 1 and 14 days of TBX-intravenous injection. These results may be attributed to the effect of biologically active, free iodine ions in CM [3]. These ionic imbalances after TBX treatments can also affect the proteins structure and functions causing several cytotoxic mechanisms such as the damage to cell membranes and mitochondrial injury leading to apoptosis and necrosis [8,9,10]. Such effects have been reported in renal cells, involving cellular energy failure, impaired calcium homeostasis, compromised tubular cell polarity and apoptosis [72,73]. Similarly, lipid peroxidation was elevated in the liver, bladder and ovary tissues by contrast media [74]. The in vivo elevated NO levels [75] and diminished GSH-Px and CAT enzyme activities were also reported [76]. The two main mechanisms, probably acting in a synergic manner, are the renal medullary ischemia and direct toxicity to renal tubular cells leading to acute kidney injury by contrast media [77,78]. Consequently, hemodynamic shifts in contrast drugs are responsible, on the one hand, for a reduction in glomerular filtration rate and, on the other hand, for medullary hypoxia in a medullary region where O_2_ supply is even low. Under normal conditions, nitric oxide (NO), prostaglandins and adenosine adjust tubular transport of sodium to adapt to this low O_2_ supply [79]. A reduced blood supply due to vasoconstriction and sodium increased reabsorption due to the elevated sodium delivery to the distal tubule will alter this mechanism, thereby causing more severe hypoxia [2].

Arabic gum has been used in Arabic folk medicine to reduce both the frequency and the need for hemodialysis in chronic renal failure patients [22]. In the current study, the supplementation of AG was expected to ameliorate the TBX-induced DNA damage and oxidative stress in kidney tissues in addition to cytogenotoxic effects in blood and bone marrow [80]. Although the current work followed the literature in the choice of AG dosing [31,32], AG itself exerted little adverse effects when compared to untreated group. However, the significant ameliorating potency of AG against the TBX toxicity was proven, further investigations of dose dependency should be performed to specify the accurate dose of AG with no adverse effects.

The majority of altered genetic conditions in humans are attributed to chromosomal aberrations [81] and may lead to some diseases as well as cancer progression [66]. The higher records of chromosomal aberrations and mitotic index in TBX-treated rats were reversed also by AG treatments in the present study. The treatment with AG demonstrated a good anticarcinogenic potential against colon carcinoma in rodents, as well as antioxidant and cytoprotective effects [82]. The elevated levels of antioxidant enzymes were evidenced and the secondary decline of oxidative stress was discussed previously for AG [83]. Similar results were recognized also for SOD and GSH levels [28]. Further, AG prevents the release of pro-inflammatory cytokines in the plasma and kidney of rats suffering from adenine-induced renal failure. Its usefulness against chronic kidney disease was owned to its anti-inflammatory and anti-oxidative potentials [28]. However, GSH plays a vital role in the elimination of reactive species [84]. AG administration prevented pulmonary inflammation and restored the lung function [85,86], by inhibiting the free radical-mediated oxidative stress and DNA damage [87]. Amino acids such as histidine, methionine and tyrosine appear to be responsible for the antioxidant properties of AG against ROS [88]. The six metabolites were identified and included flavonoids known with their excellent antioxidant activities owned to the presence of ortho-dihydroxyl groups and their role in the structure-activity relationship [89]. The flavonoid 7,3’,4’-trihydroxyisoflavone (3′-hydroxydaidzein) was mentioned before to inhibit the lipid peroxidation in the rat liver with IC50 4.1 µM [54]. It exerted also the same1,1-diphenyl-2-picrylhydrazyl (DPPH) radical-scavenging activity as α-tocopherol and similar antimutagenic activity to 6-hydroxydaidzein [90]. In case of hesperidin which was previously isolated from the same genus, it was able to significantly decrease the free radical levels in DPPH assay with similar efficacy to the positive control (Trolox) [91]. Additionally, it offers a robust cellular antioxidant protection against the damage caused by paraquat and peroxide hydrogen [92]. The opium alkaloid papaverine was found to inhibit the lipid peroxidation when given with ethanol concurrently [93]. It also demonstrated a potential protective effect against oxidative stress and its antioxidant activity on rabbit testicular tissue [94]. After the treatment with the lipophilic alkaloid noscapine in the in vitro yeast assays, a considerable antioxidant activity and improvement of the cell tolerance against oxidative stress was observed [95]. Besides, it prevents lipid peroxidation in electrochemical techniques [96]. For the first time, the sesquiterpene lactone (costunolide) was evaluated to exhibit a protective effect against oxidative stress in a study conducted by Eliza and other colleagues in 2010 [97]. Collectively, all of the identified metabolites of *A. senegal* gum were previously suggested to participate in antioxidant and protective activities. The future investigations may be recommended to compare between AG and other well-known drugs which protect against radiographic contrast medium cytogenotoxicity such as atorvastatin [98] or serofendic acid [99].

## 5. Conclusions

The present study showed the possible protective effect of Arabic gum against the genotoxicity and oxidative stress induced by Telebrix in male albino rats. The findings may be attributed to the antioxidant properties of AG which restored the oxidative status in kidney tissues and exerted anti-genotoxic potential in DNA damage and mitotic index of bone marrow. These results can be attributed to the identified metabolites of Arabic gum.

## Figures and Tables

**Table 1:** The precursor and fragments’ masses of the identified metabolites compared with that of the standards from the molecular networking database.

	Precursor Mass	Raw Mass Fragments	Library Fragments	Reference
7,3′,4′-Trihydroxyisoflavone	271.698	252.94, 242.95, 224.94, 214.93, 160.93 and 136.91	271.06, 235.05, 225.05, 215.07 and 137.02	[50]
Noscapine	414.525	352.99, 219.94 and 205.03	353.10, 220.10 and 205.07	[51]
Tetrahydropapaveroline	289.652	270.92, 242.95 and 164.02	288.12, 271.09 and 164.07	[52]
Costunolide	234.359	215.02, 187.05 and 160.05	233.15, 215.14, 187.15 and 159.12	[53]
Hesperidin	611.313	593.07, 575.01, 556.97, 488.97, 464.92, 449.02, 430.99, 413.00, 345.03 and 303.01	593.00, 575.00, 557.00, 489.00, 465.00, 449.00, 431.00, 413.00, 345.00 and 303.00	[54]

## Data Availability

Not applicable.
